# Beating the Clock: Successful Thrombectomy 28 Hours After Stroke in a Young Adult With Internal Carotid Artery Dissection

**DOI:** 10.7759/cureus.98724

**Published:** 2025-12-08

**Authors:** Bushra Qureshi, Sobiya Farook, Mohammadzakir Diwan, Sheeba Philip

**Affiliations:** 1 Department of Geriatrics, East Lancashire Hospitals NHS Trust, Blackburn, GBR; 2 Department of Emergency Medicine, East Lancashire Hospitals NHS Trust, Blackburn, GBR; 3 Department of Internal Medicine, East Lancashire Hospitals NHS Trust, Blackburn, GBR; 4 Department of Neurology, East Lancashire Hospitals NHS Trust, Blackburn, GBR

**Keywords:** acute ischaemic stroke, ct brain perfusion, internal carotid artery occlusion, stroke, stroke in young patients, stroke thrombectomy

## Abstract

Stroke is a significant diagnostic challenge in the young population. Aortic dissection is a prominent cause, among many other aetiologies, leading to subsequent neurological deficits as a result of large vessel occlusion. Neurological thrombectomy has revolutionised the management of stroke. Current clinical guidelines recommend thrombectomy within a defined time window after symptom onset. However, with advancements in modern medicine and neuroimaging, patient selection can now extend beyond the traditional time windows by identifying salvageable brain tissue.

We present the case of a young man who experienced a fall and suffered a transient loss of consciousness. He went to sleep and subsequently woke up with left-sided weakness and speech disturbances. A CT scan of the head was performed, which showed a right-sided infarct. A CT angiogram showed a right internal carotid artery (ICA) dissection complicated by occlusion of the middle cerebral artery (MCA). A CT perfusion scan highlighted a large ischaemic penumbra with a small relative infarct core, well beyond the usual therapeutic window. Given the clinical status and favourable imaging profile, even though the patient had passed the thrombectomy window, the patient underwent a mechanical thrombectomy (approximately 28 hours post onset). The procedure resulted in successful revascularisation, and there was profound neurological recovery, which was reflected in a modified Rankin Scale score (mRS) of 1 and a National Institutes of Health Stroke Scale (NIHSS) score of 1, consistent with marked clinical recovery at the time of discharge.

This case shows the importance of advanced imaging in guiding treatment decisions for patients with delayed presentation of ischaemic stroke. It underlines the significance of arterial dissection in a younger age group presenting with stroke symptoms. A thrombectomy beyond the usual norm may be a viable and effective treatment option when carefully selected in specific patients.

## Introduction

An immense diagnostic and therapeutic challenge is posed by ischaemic stroke, especially in the younger age group. Unlike in older populations, the aetiologies differ, with arterial dissection accounting for a substantial proportion of cases [1]. A well-recognised cause of ischaemic stroke is internal carotid artery (ICA) dissection, frequently resulting in neurological deficits due to large-vessel occlusion (LVO) [1].

The advent of mechanical thrombectomy has dramatically improved outcomes, particularly in LVO strokes treated within six hours of symptom onset [2, 3]. Landmark trials such as DAWN (Diffusion Weighted Imaging or CT Perfusion Assessment With Clinical Mismatch in the Triage of Wake-Up and Late Presenting Strokes Undergoing Neurointervention) and DEFUSE-3 (Endovascular Therapy Following Imaging Evaluation for Ischemic Stroke 3) have extended the therapeutic window for thrombectomy to 16-24 hours in selected patients based on advanced imaging criteria [2, 3].

The key takeaway from these studies is the shift from a time-based to a tissue-based paradigm in acute stroke management, focusing on identifying salvageable penumbral tissue even in prolonged ischaemia [2, 3]. Although the role of thrombectomy beyond 24 hours remains less well established, particularly in young patients with arterial dissection, this case report details the clinical course, imaging, treatment, and outcome of a young Romanian male with delayed diagnosed carotid dissection and middle cerebral artery (MCA) occlusion, illustrating how advanced imaging techniques can guide intervention and highlighting the potential benefits of mechanical thrombectomy even beyond traditional time windows.

## Case presentation

We present the case of a 34-year-old right-handed male patient who arrived at the emergency department on June 8, 2025, after falling from a staircase at approximately 5:30 a.m. He briefly lost consciousness and, over the course of the day, became progressively drowsy and developed slurred speech. By the evening, his family noticed weakness affecting the left side of his body.

Obtaining a detailed history and informed consent for intervention was done using certified translation services to ensure full patient understanding. His medical background included depression (managed with mirtazapine), hypercholesterolaemia, pyelonephritis, chronic back pain, and a left-sided varicocele. He smoked six cigarettes daily, drank alcohol occasionally, and admitted to using cocaine three weeks before admission.

On examination, he was alert but exhibited a left-sided facial droop, upper-limb weakness, and mild ataxia, with preserved lower-limb strength and intact sensation in both upper and lower limbs. Speech production was clear. His National Institutes of Health Stroke Scale (NIHSS) score was 6, and his pre-stroke modified Rankin Scale (mRS) score was 0.

**Figure 1:**
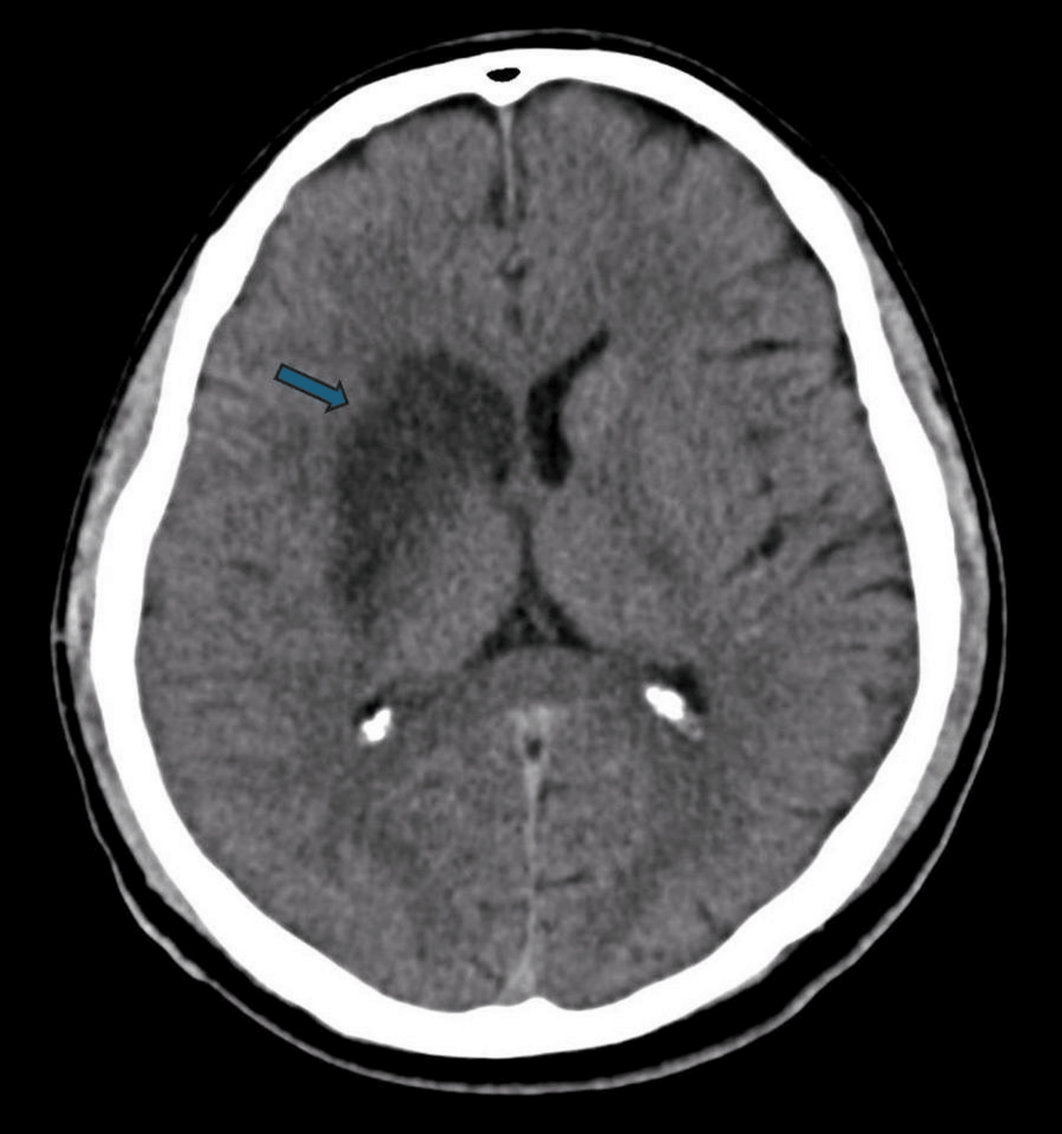
Initial non-contrast CT of the brain showing acute right middle cerebral artery territory infarct

**Figure 2:**
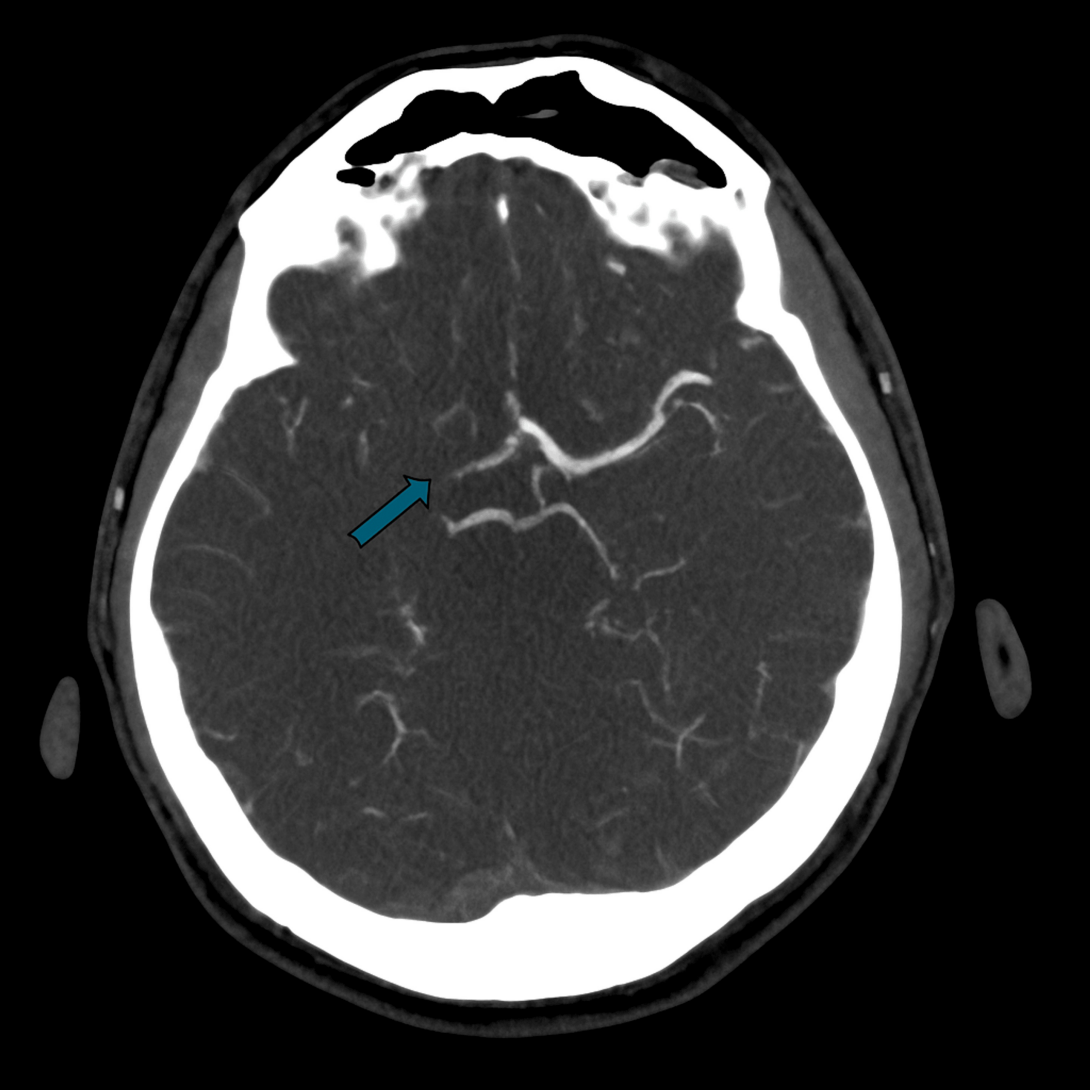
CT angiogram showing right middle cerebral artery occlusion (outlined in green).

Given his acute neurological findings, an urgent non-contrast CT of the head was performed, which demonstrated early ischaemic changes within the right basal ganglia and insular cortex, consistent with an evolving right MCA infarct (Figure 1). CT angiography revealed an irregular filling defect along the right ICA, suggesting a subacute dissection extending over 13 mm and resulting in occlusion at the carotid bifurcation and proximal MCA, findings consistent with a carotid artery dissection, a recognised cause of ischaemic stroke in young adults (Figure 2).

**Figure 3:**
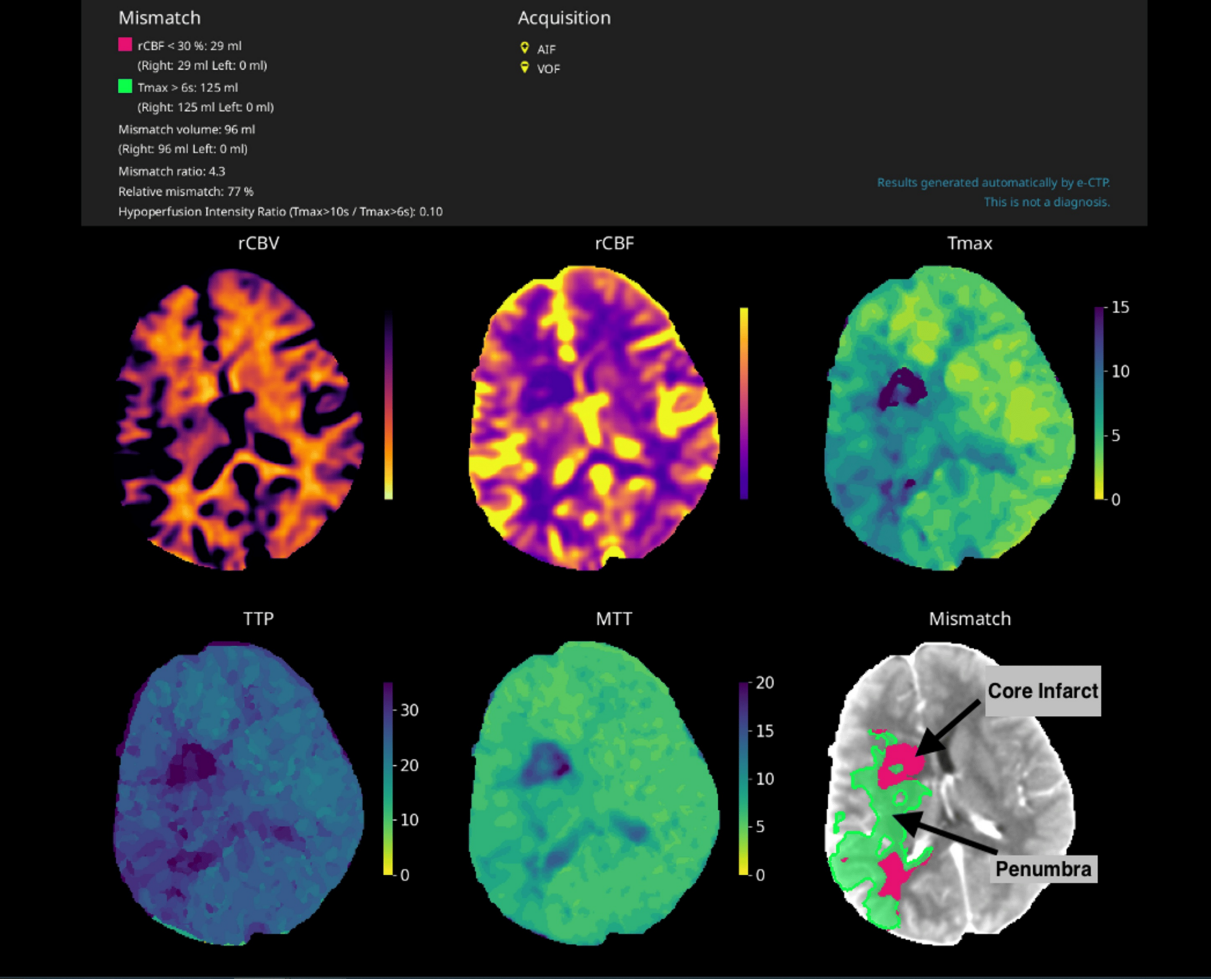
A CT perfusion scan showing the penumbra mismatch. The pink area represents the core infarct; brain tissue that has already suffered irreversible damage due to severely reduced blood flow. This tissue is not salvageable. The green area represents the ischaemic penumbra; brain tissue that is at risk but still viable if blood flow is restored quickly. This tissue is potentially salvageable with prompt treatment.

Given that symptom onset was well beyond conventional therapeutic windows, CT perfusion imaging was obtained to assess tissue viability. The scan demonstrated a large penumbra with a relatively small infarct core, indicating a significant area of potentially salvageable brain tissue (Figure 3). These results supported a decision for mechanical thrombectomy despite the extended time frame, aligning with the tissue-based approach endorsed by the DAWN and DEFUSE-3 trials [2, 3].

**Figure 4:**
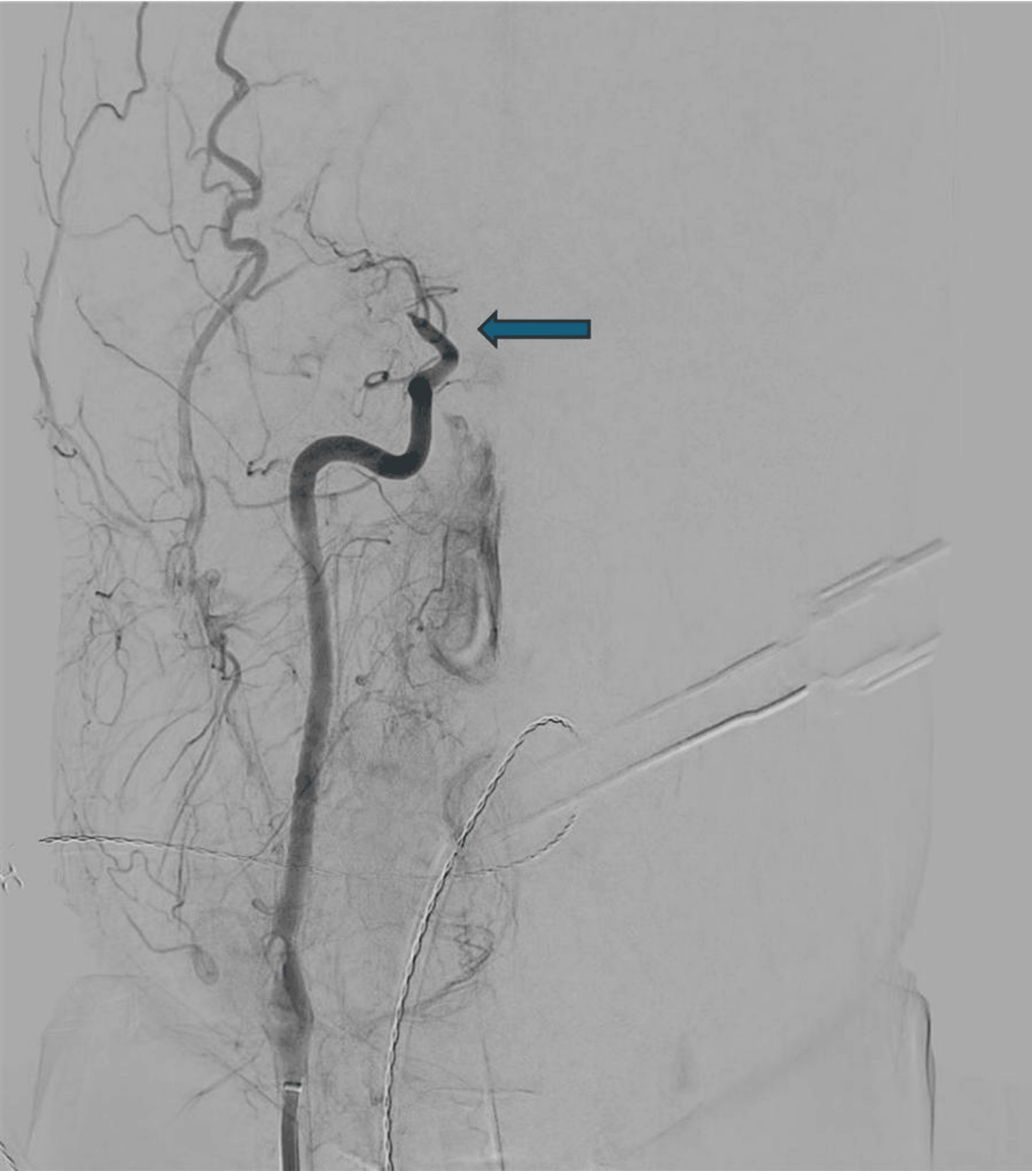
Digital subtraction angiography demonstrating proximal middle cerebral artery occlusion prior to thrombectomy.

**Figure 5:**
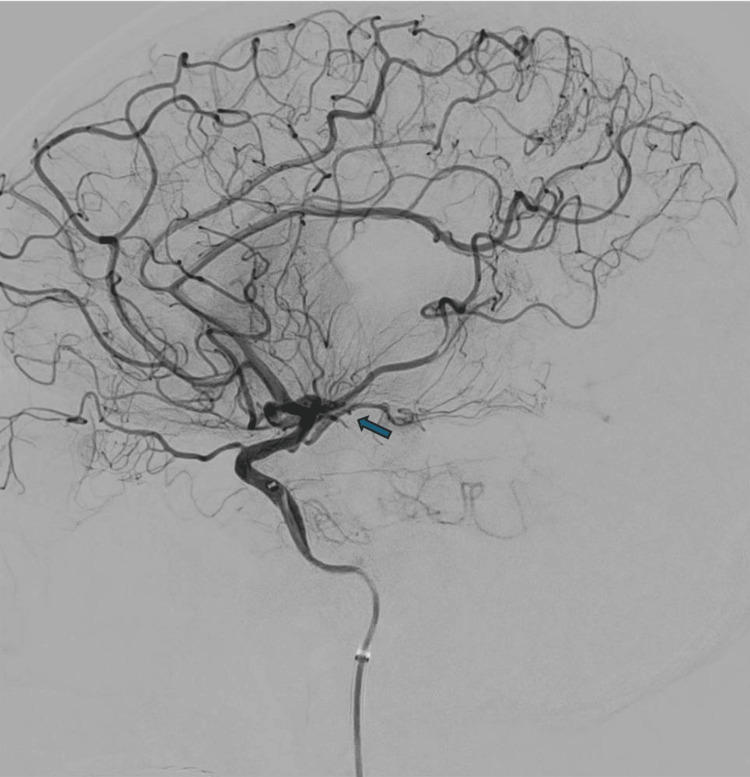
Digital subtraction angiography post thrombectomy showing complete reperfusion of the left middle cerebral artery territory.

On June 9, 2025, the patient underwent mechanical thrombectomy under general anaesthesia. Right femoral artery access was achieved, and digital subtraction angiography (DSA) confirmed occlusion of the right ICA and proximal MCA (Figure 4). Several aspiration passes were performed using SOFIA 6F (Terumo Neuro, Aliso Viejo, CA, USA), SOFIA 5F (Terumo Neuro), and the RED 43 catheter (Penumbra, Inc., Alameda, CA, USA) to achieve reperfusion. After multiple attempts, substantial revascularisation (thrombolysis in cerebral infarction (TICI) 2B) was accomplished, restoring blood flow to the affected territory (Figure 5). Haemostasis was achieved using an Angio-Seal device (Terumo Corporation, Tokyo, Japan).

**Figure 6:**
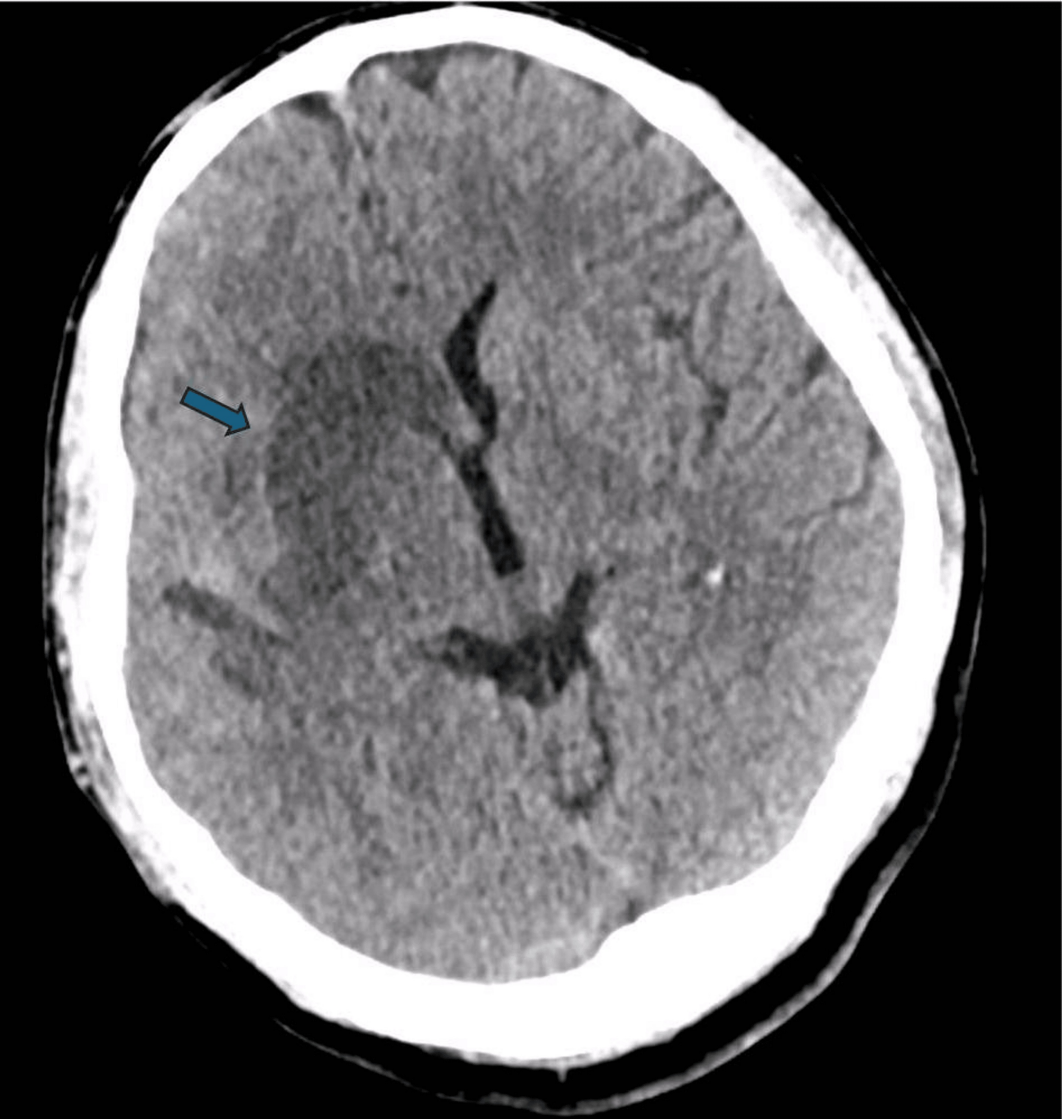
A CT scan of the head obtained 24 hours post thrombectomy showed an established infarct within the right basal ganglia and opercular region, similar to the previous CT from 09/06/25. No convincing acute haemorrhage was noted.

A follow-up CT scan 24 hours after the procedure showed a stable infarct involving the right basal ganglia and opercular cortex without evidence of haemorrhagic transformation (Figure 6). Small post-procedural hyperdensities were noted but were clinically insignificant.

Further investigations, including autoimmune markers, a thrombophilia screen, transthoracic echocardiography with bubble study, and extended ECG monitoring, were undertaken to evaluate possible stroke aetiology.

By June 11, 2025, the patient showed marked clinical improvement, regaining independent mobility and the ability to perform daily activities. Antiplatelet therapy was transitioned from aspirin to clopidogrel 75 mg on June 12, and he was discharged shortly thereafter with an mRS score of 1.

At the regional stroke multidisciplinary team review on June 16, 2025, it was concluded that thrombectomy was appropriately performed despite being outside the standard time window, as imaging had confirmed viable tissue and adequate collateral circulation. The procedure required multiple aspiration passes due to a significant thrombus load, but post-procedural imaging demonstrated minimal haemorrhagic change and preservation of right frontal lobe tissue.

## Discussion

Among individuals under 45 years, ischaemic stroke accounts for approximately 10%-15% of all cases globally [1]. In older populations, strokes are often attributed to atherosclerosis and small-vessel disease, unlike in young stroke patients, where common aetiologies include arterial dissection, cardioembolism, and prothrombotic or inflammatory disorders [1]. Arterial dissection is more prevalent in younger adults due to underlying connective tissue fragility, minor trauma, and lifestyle factors such as smoking and stimulant use. Around 20% of ischaemic strokes in this age group are caused by internal carotid artery dissection [1].

These cases necessitate a broad diagnostic approach, including vascular imaging, cardiological evaluation, and laboratory investigations such as thrombophilia and autoimmune panels. Timely management of stroke symptoms is crucial, yet diagnosis, especially in young adults, can be challenging due to atypical presentations and lower clinical suspicion. Socioeconomic and linguistic barriers may further delay diagnosis, as seen in this patient. Research indicates that younger patients often experience longer pre-hospital delays, which can influence treatment eligibility.

Advances in neuroimaging, particularly CT and MRI perfusion, have revolutionised stroke management, enabling a paradigm shift from time-based to tissue-based selection [2-4]. Landmark studies such as DAWN and DEFUSE-3 demonstrated that thrombectomy up to 24 hours after symptom onset can significantly improve outcomes compared with medical management [2,3]. Moreover, recent meta-analyses support extending thrombectomy beyond the conventional 24-hour window in selected patients with viable tissue and good collateral flow [4]. Observational data further suggest that even later interventions can yield favourable results in appropriately chosen cases [5].

Despite these encouraging results, late thrombectomy remains under investigation. Challenges include organised thrombus formation and vessel-wall pathology, which may necessitate multiple device passes and increase procedural complexity [5]. Nonetheless, young patients often demonstrate better collateral circulation and neuroplasticity, potentially leading to improved recovery even when reperfusion occurs later [5]. A multidisciplinary approach, integrating interventional, rehabilitative, and preventive strategies, is critical in optimising outcomes. Combined with psychosocial support, lifestyle modification, and long-term follow-up, these measures are essential for reducing recurrence risk and improving functional recovery.

## Conclusions

This case underscores the potential benefit of thrombectomy beyond the conventional 24-hour window in young patients with ischaemic stroke secondary to carotid artery dissection. Patient selection for thrombectomy should rely on advanced perfusion imaging rather than rigid time limits. CT perfusion and advanced neuroimaging play a pivotal role in identifying salvageable brain tissue and guiding reperfusion therapy, expanding treatment opportunities for selected patients. Further research is warranted to establish the safety and efficacy of late thrombectomy in this specific population.

Moreover, this case highlights the importance of individualised, imaging-based decision-making rather than rigid time constraints in acute stroke care. Early recognition of arterial dissection and timely access to specialised stroke centres can significantly influence outcomes, particularly in younger individuals. Continued multidisciplinary collaboration and long-term follow-up are essential to optimise functional recovery and prevent recurrent cerebrovascular events.
